# Synthesis and Pharmacological Evaluation of (6-Substituted 4-Oxo-4*H*-chromene-3 yl) methyl N-substituted Aminoacetates

**DOI:** 10.4103/0250-474X.40348

**Published:** 2008

**Authors:** Asmita Gajbhiye, V. Mallareddy, G. Achaiah

**Affiliations:** Medicinal Chemistry Research Lab, University College of Pharmaceutical Sciences, Kakatiya University, Warangal - 506 009, India; 1 Vaagdevi College of Pharmacy, Ram Nagar, Kakatiya University, Warangal - 506 009, India

**Keywords:** Chromone, bronchodilatory, antianaphylactic, asthma, antiallergic, antihistaminic

## Abstract

A series of the title compounds were synthesized and characterized by spectral data. All the compounds were evaluated for *in vitro* antihistaminic activity by inhibition of isotonic contractions induced by histamine on isolated guinea pig ileum and the compound 6-k showed significant activity. A few compounds have also been screened for *in vivo* bronchodilatory activity. These compounds exhibited significant protection against histamine-induced convulsions in guinea pig at the dose of 50 μmol.

Chromone moiety is a component of a number of biologically active substances of both synthetic and natural origin having medical significance^1^. Thus it is of great interest to medicinal chemist for molecular manipulation and pharmacological evaluation. Chromone is reported to have coronary spasmolytic^2^, bronchodilatory^3^, antiallergic^4^, antianaphylactic^4^,^5^, platelet antiaggregatory^6^ and anti-asthmatic^7^ activities. 3-(Hydroxymethyl)-4*H*-chromen-4-one is a key intermediate for the synthesis of many drugs^8^,^9^. It can be prepared by the two reported methods. The first method involves reduction of 4-oxo-4H-chromen-3-carbaldehyde using sodium borohydride in the presence of aluminum chloride and borane in THF^10^, and the second method uses condensation of 1-(2-hydroxyphenyl)-2-(methylsulfinyl)ethanone/2′-hydroxy-2-(methylsulfinyl)acetophenone with formaldehyde, followed by thermal elimination of methylsulfinyl group^11^. The first method resulted in poor yields and formation of complex mixture which required tedious purification process. Hence we followed the second method. Disodium chromoglycate, which contains chromone nucleus, is well known for prophylaxis and treatment of asthma, and on the other hand several antihistamines posses basic nitrogen. Hence it was proposed to synthesize title compounds, taking care to preserve the chromone skeleton along with the substituent in the benzo group and also to introduce a basic N-substituted amino group at the acetate group, separating the chromone ring and basic nitrogen with four atoms. Such a molecular framework is expected to retain the mast cell stabilizing potency and H_1_-receptor blockade and can be useful as both prophylactic as well as therapeutic agent in allergic patients.

Melting points were determined in open capillary tubes and are uncorrected. IR spectra were recorded on Perkin-Elmer spectrum, Bx-I IR spectrometer, ^1^H NMR on Jeol-300D (300 MHz) using TMS as internal standard and mass spectra on VG Micromass 7070H instrument. The title compounds were prepared from 3-hydroxymethyl-4-oxo-chromene^11^ as depicted in Scheme 1.

**Scheme 1:**
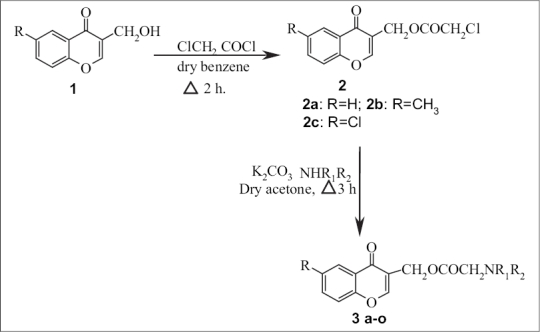
Synthesis of the title compounds-NR1R2 in which R1 is H and R2 is 2-pyridyl or morpholino or 4-methylpiperazino or 4-ethylpiperazino or piperidino

As shown in Scheme 1, the key intermediates were prepared and identified based on the reported data^10^–^13^. Compounds 2 were synthesized by refluxing appropriate 3-(hydroxymethyl)-4*H*-chromen-4-one (1; 0.01 mol) with chloroacetyl chloride (0.01 mol) in dry benzene (20 ml) under anhydrous conditions, using calcium chloride guard tube, for 2 h. The product thus formed was filtered, washed with small portions of benzene to remove unreacted chloroacetyl chloride and dried. It was purified by recrystallization from petroleum ether and benzene mixture (1:1). Infrared spectrum of the compound (2a; KBr) showed characteristic absorption frequencies at: 1641 (C = O, pyran), 1768 (C = O, ester), 2950 (C-H, aliphatic), 3069 (C-H, aromatic) cm^−1^, respectively. ^1^H NMR spectrum of the compound (2a; CDCl_3_) exhibited proton signals at (δ, ppm): 4.10 (s, 2H, -CH_2_Cl), 5.16 (s, 2H,-CH_2_OCO), 7.27-8.13 (m, 4H, C-2,C-6,C-7,C-8), and 8.23-8.26 (dd, 1H, -C-5).

Compounds 3 were synthesized from appropriate (4-oxo-4*H*-chromen-3-yl) methyl 2-chloroacetates (2; 0.01 mol) by refluxing them in dry acetone (30 ml) with appropriate amines (0.01 mol), freshly fused potassium carbonate (2.0 g) for 3 h. The reaction mixture was filtered off and residue was washed with little dry acetone. The solvent was removed under *vacuum* and the solid so obtained was purified by recrystallization using ethyl alcohol. Molecular formula of 3a C_17_H_14_N_2_O_4_ (molecular weight 310.304) requires: C: 65.80; H: 4.55; and N:9.03%; found C: 66.02; H: 4.68; N: 9.03%. Infrared spectrum of the compound (KBr; 3a; R = H) showed absorption bands at: 3284 (NH), 1670 (C = O of ester), 1654 (C = O)) and 1605 (C = N of pyridine) cm^−1^, respectively. ^1^H NMR spectrum of the compound (3a; R = H), in CDCl_3_ exhibited characteristic signals at (δ, ppm):11.57(d, 1H, NH), 8.28 (d, 1H, C-5), 6.79-8.18 (m, 8H, aromatic) and 4.97 (s, 2H, -CH_2_O), 4.98 (s, 2H, -CH_2_-N). Physical data of compounds 2a-2c and 3b-3o along with their *invitro* antihistaminic data are presented in Table 1. The compounds were screened for *in vivo* bronchodilatory activity and were subjected for toxicity studies, and found to be nontoxic and safe in experimental animals (guinea pigs) up to a dose of 300 mg/kgbw (i.p.).

**TABLE 1:** CHARACTERIZATION DATA AND *IN VITRO* ANTIHISTAMINIC ACTIVITY OF COMPOUNDS (3a-o)

Compound	R	-NR_1_R_2_	%yield	mp	% inhibition
2a	H	-	74	124-126	-
2b	CH_3_	-	85	124	-
2c	Cl	-	48	138-140	-
3a	H	R_1_ = H,R_2_ = 2-pyridyl	51	144	44.6
3b	H	Morpholino	58	98	43.1
3c	H	4-methylpiperazino	56	88	49.3
3d	H	4-ethylpiperazino	55	90	75.4
3e	H	Piperidino	56	138	49.3
3f	CH_3_	R_1_ = H,R_2_ = 2-pyridyl	88	146	55.6
3g	CH_3_	Morpholino	92	122	41.3
3h	CH_3_	4-methylpiperazino	92	112	46.8
3i	CH_3_	4-ethylpiperazino	90	129	45.2
3j	CH_3_	Piperidino	90	128	73.4
3k	Cl	R_1_ = H,R_2_ = 2-pyridyl	96	128-130	100.0
3l	Cl	Morpholino	98	106	48.4
3m	Cl	4-methylpiperazino	93	98-100	84.5
3n	Cl	4-ethylpiperazino	96	134	77.6
3o	Cl	Piperidino	97	94	94.8
Standard	-	Pheniramine maleate	-	-	96.6

Dose of test compound 60 μg; Dose of standard 50 μg

The test compounds were found to be free from CNS depression and did not cause any changes in the behavioural pattern of animals tested viz., impairment in awareness, mood and motor activity. Fifteen compounds of series 3a-o containing chromone ring attached to a basic moiety through four atom unit (-CH_2_-O-CO-CH_2_-) have been tested for their ability to inhibit histamine induced isotonic contractions in isolated guinea pig ileum (*in vitro* model). Pheniramine maleate was used as standard (50 μg; 96.6% inhibition). The results are included in Table 1.

All of them exhibited significant antihistaminic activity at the concentration of 60 μg. Of all the compounds in the series, 3k (R = Cl; R_1_ = H, R_2_ = 2-pyridyl) was found to be the most potent exhibiting 100% inhibition at the selected dose. Compound 3e with piperidine as basic moiety exhibited 49.3% inhibition and the activity increased to 94.8% with chlorine substitution at 6 position (3o; R = Cl, -NR_1_R_2_ = piperidino). Also a methyl substituent at the same position (3j; R = CH_3,_ -NR_1_R_2_ = piperidino) could cause 73.4% inhibition. However compound 3a with 2-pyridylamino group showed only 44.6% inhibition in the absence of a chlorine substituent (R = H). Thus both chlorine and methyl substituents being at 6^th^ position is contributing positively to enhance the activity. Compounds 3d and 3c with 4-alkyl piperazine as basic nucleus have been found to be more active than the compound with morpholino group (3b). As seen in case of piperidine containing compounds, chlorine substitution at 6^th^ position in compounds with peperazine also enhanced the activity.

Compounds 3k, 3n and 3o which showed greater *in vitro* potency, have been screened for *in vivo* bronchodilatory activity as well. All the three compounds exhibited significant protection against histamine induced convulsions in test animals (guinea pigs).

Among these three compounds, compound 3k (R = Cl; R_1_ = H, R_2_ = 2-pyridyl) showed relatively higher activity with 48.34% protection against 43.34% protection exhibited by standard drug (aminophylline), whereas 3o, 3n with piperidine and 4-ethylpiperazine groups showed only 38.34% and 35.99% protection respectively (Table 2).

**TABLE 2:** *IN VIVO* BRONCHODILATORY ACTIVITY OF COMPOUNDS

Compound	Mean ± standard deviation Time of onset of convulsion		%Protection
		
	Before drug treatment (T_1_) in s	After drug treatment (T_2_) in s	
Aminophylline	173.9 ± 55.1	307.0 ± 30.0	43.34
3k	189.2 ± 68.1	366.2 ± 48.4	48.34
3n	192.4 ± 51.1	300.6 ± 71.2	35.99
3o	216.6 ± 49.2	351.2 ± 32.9	38.34

Dose of test compound 50 μmol; Dose of standard 50 μmol

Selected compounds from the series (having greater *in vitro* potency) have been found to be devoid of toxicity and no mortality was observed upto a dose of 300 mg/kgbw (i.p.) in the experimental animals. Compounds 3k and 3o, which have comparable *in vitro* antihistaminic activity, differed in their *in vivo* bronchodilatory activity. Compound 3k with pyridyl moiety exhibited 48.34% protection against 38.34% protection shown by compound 3o (pyridine moiety) as compared to that of the standard aminophylline, which showed 43.34% protection.
